# On the Use of Piezoelectric Sensors in Structural Mechanics: Some Novel Strategies

**DOI:** 10.3390/s100605626

**Published:** 2010-06-03

**Authors:** Hans Irschik, Michael Krommer, Yury Vetyukov

**Affiliations:** Institute for Technical Mechanics, Johannes Kepler University Linz, Altenbergerstr.69, Linz, Austria; E-Mails: michael.krommer@jku.at (M.K.); yury.vetyukov@jku.at (Y.V.)

**Keywords:** sensor research, piezoceramics, structural control, collocation

## Abstract

In the present paper, a review on piezoelectric sensing of mechanical deformations and vibrations of so-called smart or intelligent structures is given. After a short introduction into piezoelectric sensing and actuation of such controlled structures, we pay special emphasis on the description of some own work, which has been performed at the Institute of Technical Mechanics of the Johannes Kepler University of Linz (JKU) in the last years. Among other aspects, this work has been motivated by the fact that collocated control of smart structures requires a sensor output that is work-conjugated to the input by the actuator. This fact in turn brings into the play the more general question of how to measure mechanically meaningful structural quantities, such as displacements, slopes, or other quantities, which form the work-conjugated quantities of the actuation, by means piezoelectric sensors. At least in the range of small strains, there is confidence that distributed piezoelectric sensors or sensor patches in smart structures do measure weighted integrals over their domain. Therefore, there is a need of distributing or shaping the sensor activity in order to be able to re-interpret the sensor signals in the desired mechanical sense. We sketch a general strategy that is based on a special application of work principles, more generally on displacement virials. We also review our work in the past on bringing this concept to application in smart structures, such as beams, rods and plates.

## 1. An Introductory Review on Piezoelectric Sensing and Actuation of Smart Structures

This Section gives an introductory general review on piezoelectric sensing and actuation. In the subsequent sections, we will put special emphasis on the description of some own work, which has been performed at the Institute of Technical Mechanics of the Johannes Kepler University of Linz (JKU) in the last years. These contributions have been funded in the framework of several peer-reviewed research projects listed in the Acknowledgement at the end of the paper. For other recent peer-reviewed work on piezoelectric sensing at JKU, the reader is referred to the report edited by Jakoby [1].

According to a popular definition by Jaffe [2], piezoelectricity denotes two physical effects that exist in certain materials, namely “the generation of electric charge in a substance by a mechanical stress that changes its shape, and a proportional change in the shape of a substance when voltage is applied.” A comprehensive historical exposition on the discovery of the above two piezoelectric effects was presented by Katzir [3]. Around 1880, the brothers Curie discovered the first effect, which is called the direct piezoelectric effect, to exist in certain crystals, e.g., in quartz. The Curies in 1882 also experimentally proved the second effect, called the converse piezoelectric effect, which was theoretically predicted by Lippmann shortly before. In modern applications of piezoelectricity, the direct piezoelectric effect basically is utilized for sensing, and the converse effect for the actuation of mechanical deformations. From a theoretical point of view, both techniques are based on constitutive relations, which couple the mechanical and the electrical fields. When these fields are sufficiently small, the constitutive relations can be considered as linear. For mathematical models of the piezoelectric constitutive behaviour, see e.g., Yang [4], Kamlah [5] and Niezrecki *et al.* [6]. The coupling between mechanical, electrical, thermal and magnetic fields has been discussed by Nowacki [7].

Concerning modern technological applications of piezoelectricity, Katzir [3] notes: “Though almost unknown outside the professional community, piezoelectric devices are today ubiquitous. Virtually everyone in the West posses at least one device based on piezoelectric technology.” The topic of the present paper belongs to a group of highly promising applications, namely the controlled sensing and actuation of the deformation and motion of mechanical systems. Piezoelectric materials frequently favored in this field are ferroelectric polycrystalline ceramic substances with piezoelectric behavior, so-called piezoceramics, which are known for their short response times and their high electromechanical coupling properties, such as barium titanate (BaTiO_3_) and lead zirconate titanate (PZT). A prominent example for a mass market application are piezoelectric injection systems, see Mock and Lubitz [8], where piezoceramic stack actuators are used in order to control the fuel injection in combustion engines.

The present paper is concerned with the related field of so-called smart structures. This field belongs to a part of mechatronics dealing with the overlapping areas of structural mechanics, electrical engineering and automatic control, and therefore is also called structronics or adaptronics. Structures under consideration mainly are load-carrying thin or thin-walled structural elements, such as rods, beams, plates and shells. Since they are designed for carrying loads, such as forces, thermal loadings or more complex types of eigenstrain loadings, they are also denoted as primary structures. Piezoelectric devices mostly used in connection with these structures are piezoceramic thin, flat patches, which are electroded on their upper and lower surfaces, see e.g., Miu [9]. When the two electrodes of such a patch are properly connected to the ground and to an electric voltage supply, respectively, a difference in electric potential across the patch takes place, which produces an electric field in the piezoceramic material of the patch. When the deformation of the patch is unconstrained, it then changes its geometric extensions, due to the converse piezoelectric effect. On the other hand, when this patch is deformed through an external action, such as imposed forces, temperature changes or imposed displacements, it is possible to detect an electric voltage or charge, respectively, at the electrodes, due to the direct piezoelectric effect. Now, when such a patch is firmly bonded to a surface of a primary structure, or when it is firmly integrated within a primary structure, then the patch intends to follow the deformation of the latter, despite that it is now partially constrained, and the deformation induced in the patch can be used for sensing purposes. On the other hand, when an electric field is applied to the patch due to a difference in electric voltage at its electrodes, then the patch intends to deform the primary structure, which generally leads to an actuation of the latter.

One particularly talks about a smart structure, when sensing and actuation of a primary structure via patches are coordinated by an automatic control system. The primary structure with a bonded or integrated piezoceramic patch then seemingly reacts to external disturbances like a living being. The notion of intelligent structures therefore is in use, too. For reviews on the theory and application of smart or intelligent structures, see e.g., Crawley [10], Tani *et al.* [11], Tzou [12], Rao and Sunar [13], and Liu *et al.* [14, 15]. Main applications of smart structures are the control of disturbing vibrations. In order to distinguish between passive vibration damping, one also talks about active control of structural vibrations. For a review on active structural vibration control, see e.g., Alkhatib and Golnaraghi [16]. See also the Proceedings of the World and European Conferences on Structural Control, e.g., [17–20]. In case the goal is a complete suppression of vibrations, such that the primary structure keeps its original undisturbed geometric shape everywhere and at any time, the notion of shape control is in use, see e.g., Irschik [21] for a review. This notion is sometimes also used when the displacement field of the structure is intended to follow a prescribed non-zero field of trajectories, which usually is called displacement tracking. The design of a smart structure for active vibration damping targets various multidisciplinary goals, one of them being the communication between the structure and the controller, the so-called control-structure interaction, see e.g., Gabbert and Tzou [22] and Gabbert *et al.* [23]. This notion characterizes the fact that sensors and actuators are responsible for the functioning of the interrogation and communication of the structure with the controller. Sensors provide information about the state the structure is in; this information must be interpreted and properly processed by the controller to provide the actuator with the necessary information about how to react. Concerning sensors, a discussion of strategic issues in the sensor design has been given by Liu *et al.* [15]. Frontiers in sensors/sensor systems have been described by Glaser *et al.* [24]. In typical continuous smart structures, a crucial point is how the locations of the sensors are distributed throughout the structure in order to obtain an information that is suitable for successfully performing control of vibrations of continuous structures, see Gabbert and Tzou [22]. Particularly, Lee and Moon [25] introduced the concept of shaping the electrode pattern of a continuous piezoelectric film consisting of a polymeric piezoelectric substance to measure some specific modal amplitude of a vibrating structure; such sensors with the corresponding special spatial distribution are denoted as modal sensors. With respect to the application of modal sensors, see e.g., Tzou and Hollkamp [26] and Sun and Tong [27]. For a recent formulation concerning distributed sensors realized by piezoelectric films, see Preumont *et al.* [28]. It is to be noted that the classical realization of thin films in the form of piezoelectric polymers, mostly Polyvinylidene Fluoride (PVDF), show a comparatively low electromechanical coupling behavior, and thus is mainly used for sensing purposes. When piezoceramic materials are used, however, continuous sensor distributions so far in practice have to be lumped into a dense network consisting of a large number of sensor patches. Piezoceramic strips or fiber nets embedded into a polymeric matrix are promising candidates for the next generation of powerful spatially distributed sensors and actuators. It is also possible to use piezoceramics for both sensing and actuation, as in the case of self-sensing actuators. The idea of self-sensing actuators was proposed by Dosch *et al.* [29] and Anderson *et al.* [30]. For more recent contributions, see e.g., Giurgiutiu and Zagrai [31] and Y. Nam *et al.* [32].

Generally, there is an agreement in the international scientific engineering community that smart sensor systems consisting of continuously distributed sensors or dense networks of sensor patches will enhance the way structural systems are monitored for increasing their structural safety in several respects, not only concerning active control of vibrations. Overviews on research in Europe and the USA on smart sensor systems, particularly concerning the application to structures of civil and mechanical engineering, were e.g., presented in the Proceedings of joint ESF-NSF workshops, see Faravelli and Spencer [33] and Shoureshi [34]. Particularly, a lack of a computational framework on which to build new strategies for realizing massively distributed smart sensors has been noted in a review by Spencer *et al.* [35] on opportunities and challenges in smart sensor technology.

Some critical issues of the above shortly sketched smart structures technology have motivated the work of our group. First, the functioning of smart structures is intrinsically connected to the specific automatic control methodology under consideration. Model based automatic control seems to be most promising, which means that suitable physics based mathematical models of structural mechanics and electrodynamics are needed, see Kugi [36]. For comprehensive discussions on various models used in vibration control of active structures, see e.g., Preumont [37]. It is well known that under certain circumstances a neglected system dynamics in the model under consideration may lead to an undesired excitation of the structure, leading to an unwanted contribution to the sensor signal in the control loop. The performance of the smart structure then can be degraded, or even can be destabilized. The possibility of this type of failure has been observed already at early stages of the development of structural control, and is known as spill-over, see Balas [38]. It was found that spill-over can be resolved by using a collocated actuator/sensor pairing, Meirovitch and Baruh [39] and application of advanced methods Meirovitch [40]. Structural control without collocation needs the application of advanced methods, see e.g., Meurer *et al.* [41] for a recent contribution. Without going into the details of the mathematically rather involved topic of collocated actuation and sensing, it can be said that special emphasis should be laid upon the flow of energy between structure, sensors, actuators and controller, see Kugi [36] and Ortega *et al.* [42]. In terms of mechanics, the inputs provided by the actuators and the outputs measured by the sensors should form work-conjugated pairs. This in turn brings into the play the question of piezoelectrically sensing mechanically meaningful structural quantities, such as displacements, slopes, or more general quantities, which form the work-conjugated quantities of the actuation. Such a question of course has a practical importance in its own right, apart from active control of structural vibrations. Now, at least in the range of small strains, there is generally confidence that distributed piezoelectric sensors or sensor patches in smart structures do measure weighted integrals over their domain, see e.g., Miu [9], Preumont [37], Sirohi and Chopra [43]. Therefore, there is a need of distributing or shaping the sensor activity in order to be able to re-interpret the sensor signals in the desired mechanical sense, as a displacement, slope, etc. A general strategy, which has been followed by our group, and which is based on a special application of work principles, more generally on displacement virials, is sketched in Section 2 below. In Section 3, we review our work in the past on bringing this concept to application in smart structures, such as beams, rods, and plates. We thereby review also own contributions on the resolution of other critical issues of smart structures technology, such as the questions of accurately describing the mechanical and electrical fields in the piezoelectric sensors and actuators in a manner that fits to mathematical models of structural mechanics. Here, the coupling between the direct and the converse piezoelectric effect has to be taken into account, as well as the fact that modern piezoceramics, in contrast to piezopolymers, have a considerable own stiffness and mass.

## 2. A General Strategy for Distributing Sensors such that the Output can be Interpreted as Displacement Virial or Elementary Work

In order to bring into the play the notion of work, which is the scalar product of a force system and conjugate displacement entities, see e.g., Gurtin [44], consider an auxiliary static problem of the body, with an auxiliary static stress field ***σ̄***, the body being subject to a field of externally applied body forces **b̄**. An overbar indicates quantities of the auxiliary problem. The local form of the equilibrium conditions for the auxiliary problem reads:

(1) $$\nabla \cdot \bar{\sigma }+\bar{b}=0$$

In general, both the static auxiliary stresses and the auxiliary body forces are functions of the place **x** within the body:

(2) $$\bar{\sigma }=\bar{\sigma }(x),\;\;\bar{b}=\bar{b}(x)$$

Now consider an actual vibration of the body, which is intended to be measured. In contrast to the auxiliary static problem, the actual problem may be dynamic, such that the actual displacement field is transient,

(3) u=u(x,t)

where *t* denotes time. We manipulate the auxiliary equilibrium relation in Equation (1) by a scalar multiplication with the transient actual displacement field **u** in Equation (3). The following tensorial identity

(4) $$(\nabla \cdot \bar{\sigma })\cdot u=\nabla \cdot ({\bar{\sigma }}^{T}\cdot u)-\nabla u\cdot \cdot \bar{\sigma }$$

is taken into account. We integrate over the volume *v* of the body. Using the divergence theorem, the result is

(5) $$\int_{s}({\bar{\sigma }}^{T}\cdot u)\cdot n\mathrm{ds}-\int_{v}\nabla u\cdot \cdot \;\bar{\sigma }\mathrm{dv}+\int_{v}\bar{b}\cdot u\mathrm{dv}=0$$

The outer unit normal vector at the surface *s* of the volume *v* is denoted as **n**. Noting that

(6) $$({\bar{\sigma }}^{T}\cdot u)\cdot n=(\bar{\sigma }\cdot n)\cdot u$$

and taking into account Chauchy’s fundamental theorem on stresses

(7) $${\bar{t}}_{n}=\bar{\sigma }\cdot n$$

where **t̄***_n_* denotes the auxiliary traction at the surface *s*, Equation (5) can be written as

(8) $$V_{\left(-,a\right)}(t)=\int_{v}\nabla u\cdot \cdot \bar{\sigma }\mathrm{dv}$$

where the displacement virial of the external auxiliary forces done on the actual displacements is

(9) $$V_{\left(-,a\right)}(t)=\int_{s}{\bar{t}}_{n}\cdot u\mathrm{ds}+\int_{v}\bar{b}\cdot u\mathrm{dv}$$

For the notion of displacement virials, see e.g., Irschik [45]. Since we have integrated over the volume *v* of the body at time *t*, the virial *V*_(−_*_,a_*_)_(*t*) is a function of *t* only. The actual displacements u in Equation (9) need not to be small. In case of infinitesimal actual displacements, however, *V*_(−_*_,a_*_)_(*t*) represents the elementary work of the external auxiliary forces upon the actual displacements. For the notion of elementary work, see e.g., Ziegler [46]. If one utilizes concentrated actions of unit amount for the external forces, which mathematically can be described by singular distributions, such as Dirac functions or their derivatives, then *V*_(−_*_,a_*_)_(*t*) represents the work conjugated actual displacements, slopes or relative displacements at the locations of the concentrated actions. In any case, *V*_(−_*_,a_*_)_(*t*) has a strong physical interpretation. The question therefore arises, whether *V*_(−_*_,a_*_)_(*t*) can be measured by suitable distributed sensors or by networks of sensor patches. Our group has presented answers to this question, which, although it appears to be important from several points of view, has not been in the mainstream of research in the past. A review on our results will be presented in the next Section.

It may be interesting to note that the above derivation holds in both, the Euler and the Lagrange description of continuum mechanics, and such holds also for large displacements and displacement gradients. In the Euler formulation, **x** denotes the actual place of the particle, ***σ̄*** is a Cauchy stress tensor, and n is the unit outer normal vector of the actual surface *s*. In the Lagrange description, **x** denotes the place of the particle in the reference configuration, ***σ̄*** is a first Piola Kirchhoff stress tensor, and n denotes the unit outer normal vector of the surface *s* in the reference configuration. Recall that the first Piola Kirchhoff stress tensor is not symmetric, in contrast to the Cauchy stress. Despite the auxiliary problem refers to static entities, while from the actual problem only the displacement does come into the play, we are not allowed to mix these two formulations by applying them to the auxiliary and the actual problem differently, since in Equation (4) the nabla operator ∇ must be used consistently, either with respect to the place in the actual or in the reference configuration. Integrations in Equations (8) and (9) are correspondingly to be performed over the volume in the actual or in the reference configuration, respectively. When displacements and displacement gradients are small, in the geometrically linearized theory, which is also denoted as the small strain or small deformation regime, both descriptions become coincident. The stress then is symmetric,

(10) $$\bar{\sigma }={\bar{\sigma }}^{T}$$

such that we may write

(11) $$\nabla u\cdot \cdot \bar{\sigma }=\mathrm{sym}\nabla u\cdot \cdot \bar{\sigma }$$

Now, the symmetric part of the actual displacement gradient tensor represents the linear or small strain tensor, see Gurtin [44] and Ziegler [46]:

(12) ε=ε(x,t)=sym∇u

Hence, instead of Equation (8) we can write

(13) $$V_{\left(-,a\right)}(t)=\int_{v}\varepsilon \cdot \cdot \bar{\sigma }\mathrm{dv}$$

As discussed in Section 1 above, there is confidence in the literature that, in the small strain regime, piezoelectric patches in smart structures measure weighted integrals over the actual strains, such that their output in the present context can be basically written as

(14) $$y(t)=\int_{v}\varepsilon (x,t)\cdot \cdot S(x)\mathrm{dv}$$

where **S**(**x**) denotes a matrix of spatially distributed shape functions, which contains the electromechanical material parameters. Now, if we choose

(15) $$S(x)\;\;=\bar{\sigma }(x)$$

then the output of the sensor is

(16) $$y(t)=V_{\left(-,a\right)}(t)$$

This attaches a strong physical meaning to the sensor output, in the framework of the notions of elementary work and work-conjugacy.

Some further remarks seem to be in order. First, in case of a limited region of sensing activity in the body, e.g., for a single patch, the above volume *v* must coincide with this region, and the auxiliary problem must represent an equilibrium problem for that part. Moreover, when a part of the surface *s* of *v* is a fixed boundary, then the auxiliary tractions **t̄***_n_* do not contribute to the elementary work *V*_(−*,a*)_(*t*) on this part of *s*. We then talk about workless tractions. Moreover, the boundary conditions at *s* in the auxiliary and the actual problem need not to coincide. Now assume that all of the auxiliary tractions **t̄***_n_* are workless, because the actual displacements do vanish at *s*, or that the auxiliary tractions themselves do vanish there, because the boundary of the auxiliary problem is taken as traction free:

(17) $$s:\;\;\;{\bar{t}}_{n}=0\;\;\;\;\;\mathrm{or}\;\;\;\;u=0$$

and that there are no auxiliary body forces,

(18) $$v:\;\;\;\;\bar{b}=0$$

then the virial in Equation (9) vanishes, and the auxiliary stress is divergence-free, see Equation (1):

(19) $$\nabla \cdot \bar{\sigma }=0$$

A non-vanishing auxiliary stress that satisfies Equation (19) can be produced by eigenstrains, such as a temperature loading. Hence, if we choose the matrix of shape functions according to Equation (15) under the conditions of Equations (17) and (19), then the output of the sensor vanishes for every non-vanishing actual displacement field **u**(**x**, *t*). For such a strain sensor, we have introduced the notion of a nilpotent sensor. Note that nilpotent sensor distributions can be added to a sensor measuring elementary work, without changing the output of the sensor.

In summarizing, we can say that a strain-type sensor with an output described in the form of Equation (14) measures the elementary work done by an external auxiliary force system upon the actual displacement field, if the shape matrix is chosen according to Equation (15), see Equation (16). Nilpotent sensor distributions can be identified and added. If and how this concept can be transferred from the above three-dimensional formal derivation to smart structures, such as beams, plates and shells, has been a research topic of our group in the last years, which will be shortly reported below. The question of an application in case of moderately large or even large deformations will also be addressed subsequently.

## 3. A Short Review on Own Contributions on Smart Sensing and Actuation

We start with contributions concerning accurate electromechanical modeling of smart structures, taking into account the coupling between the direct and the converse piezoelectric effect. In short, if an electric field is produced due to some deformation in the piezoelectric material, this field may have an actuating effect also, and thus in turn may influence the mechanical response of the structure. While these effects are usually of different order of magnitude, they may not be neglected in an accurate modeling. Our goal here has been to present formulations that allow circumventing the solution of three-dimensional (3D) partial differential equations of electrostatics by incorporating the coupling effects into equivalent single layer 1D or 2D theories for smart structures. The influence of the electric field on free transverse vibrations of smart beams was treated by Krommer and Irschik [47]. A Reissner-Mindlin-type plate theory including the direct piezoelectric and the pyroelectric effect was presented in [48]. An electromechanically coupled plate theory taking into account the influence of shear, rotatory inertia and electric field was given by Krommer in [49]. Further advanced studies, partly having non-local constitutive relations, were presented by Krommer in [50–54]. These studies resulted in equivalent single layer theories with effective stiffness terms and effective piezoelectric actuation terms, which can be directly computed from the electric boundary conditions under consideration.

We continue by referring some of our contributions concerning dynamic shape control of smart structures by piezoelectric actuation and sensing. In [55, 56], we have systematically treated dynamic shape control in the framework of forced vibrations of smart elastic beams, where we have found the following solution: Assume that the beam motion starts from rest and is produced by an imposed system of forces separable in space and time, with a given spatial distribution and a given time-evolution. Then it is sufficient to compute the quasi-static bending moments and, if they are involved, shear-forces, normal forces and torsional moments due to the imposed spatial force distribution. The piezoelectric actuation is spatially shaped exactly according to the latter quasi-static entities, and the piezoelectric actuation is assigned with the negative of the time-evolution of the forces. Then, no displacements are produced in total by the forces and the piezoelectric actuation, such that a complete annihilation of the force-induced vibrations is obtained. Moreover, we were able to identify inappropriate piezoelectric actuator and sensor shapes that are not able to actuate any deformation, see the complementary nilpotent sensors mentioned in Section 2 above. Important for the present context of piezoelectric sensing, we have studied several quite complex effects in [55–63], such as sensor distributions that are collocated to the actuator shapes, general classes of nilpotent sensors, self-sensing actuators, shear-actuation, and, last but not least, superimposed rigid-body motions. Solutions for bending of smart plates, extending the above studies on beams, were presented by Nader *et al.* in [64]. Automatic control of force-induced vibrations of circular plates was studied by Gattringer *et al.* in [65]. The findings of the above mentioned beam studies concerning collocated sensing were extended to flexure of plates. A new branch of shape control, namely to suppress displacements in sub-domains of force-loaded smart beams and plates, was successfully studied by Krommer and Varadan in [66–68] and Krommer in [69, 70]. A fundamental part of our studies was concerned with dynamic shape control in the framework of the three-dimensional linear theory of elasticity in the presence of piezoelectric sensors and actuators. Concerning piezoelectric actuation, these studies extended previous static results derived by Irschik and Ziegler in [71]. For dynamic problems, they were based on preliminary analogous formulations for thermal actuation by Irschik and Pichler in [72], see the more general formulation by the same authors in [73]. In the sense of the notion of work conjugacy, the results are complementary to the sensing results presented in Section 2 above: Assume the body to be loaded by transient forces, the motion starting from rest. Assume that the forces are separable in space and time. Compute the static stress tensor due to the spatial force distribution only, and shape the actuation accordingly throughout the body. Apply the negative time-evolution of the forces to the actuation. Then, the total displacements vanish everywhere at all times. In [72, 73], inappropriate actuator distributions, which do not produce displacements, were identified as stress distributions due to any imposed eigenstrain distribution, e.g., due to a temperature loading.

In extension of our studies on shape control, *i.e.,* on the suppression of vibrations produced by imposed forces in smart structures, we have studied the problem of producing desired displacement fields by piezoelectric actuation. This problem is also called displacement tracking problem. A first three-dimensional solution for determining suitably distributed actuators was presented in [74]. In the latter reference, visco-elastic material behavior was taken into account, involving the general Boltzmann convolution formulation. Various piezioelastic structural applications were treated in [75–79]. The actuator formulations in [72–79] were systematically brought together with the sensor formulation sketched in Section 2 above by Krommer and Irschik in [80]. Distributed strain type sensors and actuators were considered, and the concept of collocated sensors and actuators and the so-called natural output was introduced in a three-dimensional framework. The principle of virtual work was used to assign a mechanical interpretation to the natural output of the sensors to be designed. An extended body force analogy was used to assign a mechanical interpretation to the collocated actuators as well. The proposed sensors and actuators then were applied to solve the displacement tracking problem for smart structures, where feed forward and feed back control were discussed, and a clamped-clamped beam was studied in more detail. In the case of feed back control it was shown that a PD controller can stabilize the smart structure. Desired deflections were tracked by means of feed forward control, feed back control and a combination of the two.

Interesting numerical as well as experimental verifications on structural control by smart actuation are to be found in the doctoral thesis by Pichler [81]. Advanced methods of feed forward and feed back control of smart structures with the goal of compensating vibrations in smart structures were developed in the doctoral thesis of Nader [82], in which experimental realizations were presented, and the application to the suppression of structure borne noise was discussed, see also Nader *et al.* [83] for the latter topic. For a review of the work of our group on smart actuation in the light of mechatronics, which contains a more detailed description of noise suppression, see [84].

The studies presented above gave raise to further questions and problems, as it should be. We shortly mention the following recent results of our group. The necessity of using plate theory in wide beam-type structures has been demonstrated by Huber *et al.* in [85, 86]. Krommer *et al.* [87] studied strain-type sensor networks for structural monitoring of beam-type structures, where they presented integral equation based methods for accurately replacing continuous sensors by sparse sensor networks made of piezoceramic patches. The methods developed in [87] have been further applied to structural monitoring of multi-storey frame structures by Krommer and Zellhofer in [88]. In extension to using piezoelectric layers for sensing only, Schöftner and Irschik used shaped piezoelectric layers for passive damping and exact annihilation of beam vibrations in [89] with promising further applications to effective energy harvesting. In analogy to the methods developed for designing sparse sensor networks, methods for designing actuator networks have been reported in [90, 91]. Recently, a new direction of research has been opened by Krommer and Vetyukov in [92], who studied piezoelectric sensing of kinematically meaningful entities in the vicinity of a time-dependent geometrically nonlinear pre-deformed state. This paper represents a successful account of designing continuous strain-type sensors that can measure mechanically meaningful structural entities, such as displacements, slopes and relative displacements, which belong to small motions superposed upon a finite pre-deformation. Using the Lagrange description of continuum mechanics, it has been shown that the required sensor distribution can be computed from an auxiliary static problem similar to the procedure sketched in Section 2 above. The method then has been applied to nonlinear rod structures, and adaptive sensor distributions have been presented, for which the distribution is changed in time in order to account for time-dependent rod reference configurations. As a possible issue for future research, placement and adaption of sensor networks consisting of discrete piezoceramic patches in extension of the linear results presented by Krommer *et al.* in [87] has been mentioned. A structural application of sensing in the vicinity of large static pre-deformations of geometrically nonlinear shells was suggested by Krommer and Vetyukov in [93].
